# Environmental Enrichment and Gut Inflammation Modify Stress-Induced c-Fos Expression in the Mouse Corticolimbic System

**DOI:** 10.1371/journal.pone.0054811

**Published:** 2013-01-17

**Authors:** Florian Reichmann, Evelin Painsipp, Peter Holzer

**Affiliations:** Research Unit of Translational Neurogastroenterology, Institute of Experimental and Clinical Pharmacology, Medical University of Graz, Graz, Austria; Kaohsiung Chang Gung Memorial Hospital, Taiwan

## Abstract

Environmental enrichment (EE) has a beneficial effect on rodent behaviour, neuronal plasticity and brain function. Although it may also improve stress coping, it is not known whether EE influences the brain response to an external (psychological) stressor such as water avoidance stress (WAS) or an internal (systemic) stressor such as gastrointestinal inflammation. This study hence explored whether EE modifies WAS-induced activation of the mouse corticolimbic system and whether this stress response is altered by gastritis or colitis. Male C67BL/6N mice were housed under standard or enriched environment for 9 weeks, after which they were subjected to a 1-week treatment with oral iodoacetamide to induce gastritis or oral dextran sulfate sodium to induce colitis. Following exposure to WAS the expression of c-Fos, a marker of neuronal activation, was measured by immunocytochemistry. EE aggravated experimentally induced colitis, but not gastritis, as shown by an increase in the disease activity score and the colonic myeloperoxidase content. In the brain, EE enhanced the WAS-induced activation of the dentate gyrus and unmasked an inhibitory effect of gastritis and colitis on WAS-evoked c-Fos expression within this part of the hippocampus. Conversely, EE inhibited the WAS-evoked activation of the central amygdala and prevented the inhibitory effect of gastritis and colitis on WAS-evoked c-Fos expression in this region. EE, in addition, blunted the WAS-induced activation of the infralimbic cortex and attenuated the inhibitory effect of gastritis and colitis on WAS-evoked c-Fos expression in this area. These data reveal that EE has a region-specific effect on stress-induced c-Fos expression in the corticolimbic system, which is likely to improve stress resilience. The response of the prefrontal cortex – amygdala – hippocampus circuitry to psychological stress is also modified by the systemic stress of gut inflammation, and this interaction between external and internal stressors is modulated by the housing environment.

## Introduction

Environmental enrichment (EE) has several beneficial effects on rodent behaviour and brain neurochemistry [1]. Most notably, EE improves spatial learning and memory [1]–[3], while the effects of EE on exploratory and emotional-affective behaviour are less consistent [4]–[9]. The behavioural changes induced by EE are accompanied by morphological and neurochemical alterations within the brain [1], [10] such as increased neurogenesis [11], [12], enhanced growth factor gene expression [13]–[15] and increases in neurotransmitter levels [16].

Given that EE enhances neuronal plasticity, it has been argued that EE improves the welfare of captive animals as it enables them to express a much greater variety of species-specific behaviours than animals maintained under standard laboratory housing conditions [1]. If so, the question arises as to whether EE can improve the coping with external stressors such as psychological stress or internal stressors such as inflammation. Indeed, some studies indicate that EE can lower the response of rodents to psychological stress [4], [6], [17], [18] and improve their recovery from this adverse condition [19]. These implications have been revealed by measuring key components of the hypothalamic-pituitary-adrenal (HPA) axis. Thus, EE is able to blunt the rise of adrenocorticotropic hormone (ACTH) and corticosterone plasma levels in response to individual housing stress [17], exposure to cat odour [4] or restraint stress [18], [20].

In contrast to external stressors, little is known as to whether EE has any effect on the brain responses to internal stressors such as inflammation, except that inflammation-induced pain responses may be altered. A report by Tall [21] shows that the thermal hyperalgesia caused by injection of Freund's complete adjuvant into the hindpaw is reduced in rats prehoused under enriched conditions. Similarly, EE accelerates recovery from the mechanical allodynia induced by carrageenan-induced inflammation of the rat knee [22]. In contrast, Shum et al. [23] report that EE does not alter acute thermo- and chemonociception in mice but enhances the delayed nociceptive response to subcutaneous injection of formalin and increases the delayed mechanical allodynia evoked by Freund's complete adjuvant. However, the effect of EE on visceral inflammation and brain responses to visceral inflammation has not yet been investigated.

While EE modifies particular reactions to external and internal stressors, it remains unclear whether they reflect a change in the stress responsiveness of pertinent neuronal circuits in the brain. It was hence the aim of the current study to investigate in which way EE modifies psychological stress-induced activation of autonomic and corticolimbic brain areas and whether this cerebral stress response is altered by gastrointestinal inflammation. In addition, it was examined whether EE and inflammation alter the stress-induced release of corticosterone and whether EE has an impact on gut inflammation.

Stimulation of central neurons was visualized by immunocytochemical expression of c-Fos, an established marker of neuronal activation [24]. The experimental interventions under study were the water avoidance stress (WAS) paradigm as a model of psychological stress and both iodoacetamide (IAA)-induced gastritis and dextran sulfate sodium (DSS)-evoked colitis as models of internal stress. Like WAS [25], IAA-induced gastritis [26], [27] and DSS-induced colitis [28], [29] have been shown to cause mechanical and chemical hyperalgesia in the rodent gut. In addition, WAS and experimental colitis are known to interact with each other in a complex manner [30]–[35], given that WAS can enhance the permeability of the intestinal mucosa [36], [37]. In a translational perspective, the current study was designed to shed light on the impact of environmental factors such as stress on gastritis, inflammatory bowel disease and irritable bowel syndrome [38]–[40].

## Materials and Methods

### Experimental animals

The study was carried out with 80 adult male C57BL/6N mice obtained from Charles River (Sulzfeld, Germany). The mice were housed under controlled conditions of temperature (set point 21°C) and air humidity (set point 50%) and under a 12 h light/dark cycle (lights on at 6∶00 h, lights off at 18∶00 h). All experiments were approved by an ethical committee at the Federal Ministry of Science and Research of the Republic of Austria (BMWF-66.010/0037-II/10b/2008 and BMWF-66.010/0073-II/10b/2009) and conducted according to the Directive of the European Communities Council of 24 November 1986 (86/609/EEC). The experiments were designed in such a way that both the number of animals used and their suffering was minimized.

### Experimental protocols

Two different protocols were used. In *protocol 1*, one group of 10 mice was kept under standard housing whereas another group of 10 mice was maintained under enriched housing for 9 weeks (week 1–9). During week 10 the animals were subjected to the Morris water maze test. In *protocol 2*, 3 groups of 10 mice each were housed under standard conditions while 3 other groups of 10 mice each were housed under enriched conditions for 9 weeks (week 1–9). During week 10 the animals were subjected to different treatments, during which the housing under standard or enriched conditions was continued. Mice were treated either with IAA (0.1%) or DSS (2%) added to the drinking water, whereas the control animals drank plain water. One day after the 7-day treatment period (week 11, day 1) the animals were exposed to WAS for 30 min. Following a 90-min stress-free interval in the home cage, the animals were euthanized with an overdose of pentobarbital (150 mg/kg injected intraperitoneally). Trunk blood was collected for measurement of corticosterone, the stomach and colon were excised for determination of the myeloperoxidase (MPO) content, and the brain was collected for immunocytochemical visualization of c-Fos expression in selected brain regions.

### Standard and enriched housing conditions

Under *standard conditions* mice were housed in groups of 5 in polycarbonate cages of size IIL measuring 36.5×20.7×14.0 cm (length x width x height, floor area: 530 cm^2^). Under *enriched conditions* mice were kept in groups of 5 in polycarbonate cages of size IV measuring 59.0×38.0×20.0 cm (length × width × height, floor area: 1815 cm^2^) [1], [4], [41]. Thus, under standard conditions an average floor area of 106 cm^2^ was available to each animal, compared with a floor area of 363 cm^2^ per animal under enriched conditions.

In addition, the following enrichment items were provided [42]–[44]: nesting material (standard paper towels), a running wheel (diameter: 14 cm; Dehner, Graz, Austria), a tunnel made of hay (length: 20 cm, inner diameter: 5–8 cm; Dehner, Graz, Austria), a tunnel made of timber with 6 side holes (length: 25 cm, inner diameter: 4 cm; Dehner, Graz, Austria), a tunnel made of cardboard (length: 13 cm, inner diameter: 9 cm; Scanbur, Karlslunde, Denmark), mouse houses with two openings made of red transparent polycarbonate (10×9×5.5 cm (length × width × height; Ehret, Tulln, Austria), mouse houses with 8 openings made of cardboard (17×17×7 cm (length × width × height; Scanbur), and a tunnel made of red transparent polycarbonate (length: 10 cm, inner diameter: 5.5 cm; Scanbur) which during certain weeks of the enriched housing procedure was hung on the cage lid (Table 1).

**Table 1:** Enrichment protocol.

Week	Experimental Phase	Basic items	Additional houses	Additional tunnels
Week 1	Basic Enrichment	Nesting material, hay tunnel, running wheel	2P	1T
Week 2	Basic Enrichment	Nesting material, hay tunnel, running wheel	2P	1T
Week 3	Basic Enrichment	Nesting material, hay tunnel, running wheel	2P	1T
Week 4	Basic Enrichment	Nesting material, hay tunnel, running wheel	2P	1T
Week 5	Enforced Enrichment	Nesting material, hay tunnel, running wheel	1P+1H	1H
Week 6	Enforced Enrichment	Nesting material, hay tunnel, running wheel	2H	1T+1H
Week 7	Enforced Enrichment	Nesting material, hay tunnel, running wheel	2P	1T+1P*
Week 8	Enforced Enrichment	Nesting material, hay tunnel, running wheel	2H	1T+1P
Week 9	Enforced Enrichment	Nesting material, hay tunnel, running wheel	1P+1H	1P+1P*

* =  Tunnel hung on cage lid; H  =  hard paper; P  =  polycarbonate; T  =  timber.

Throughout the 9 week enrichment procedure, nesting material, a running wheel and a hay tunnel were available (Table 1). At the beginning of each week the mice were placed in a clean cage with fresh bedding; the enrichment items were also thoroughly cleaned or provided fresh. In addition, two polycarbonate mouse houses and a timber tunnel were offered during the first 4 weeks. From week 5 onwards, the mouse houses (polycarbonate or hard paper) and tunnels (timber, polycarbonate or hard paper) were exchanged every other week as shown in Table 1.

### Induction of experimental gastritis or colitis

Iodoacetamide (IAA, Sigma, Vienna, Austria) was added to the drinking water at a concentration of 0.1% (w/v) for a period of 7 days to induce mild gastritis [45]. The control animals received normal tap water. Since IAA is light-sensitive, the IAA-containing drinking water was made up fresh every day [45].

Mild colitis was induced by adding dextran sulfate sodium (DSS, molecular weight 36,000–50,000; MP Biomedicals, Illkirch, France) at a concentration of 2% (w/v) to the drinking water for 7 days [29]. The control animals received normal tap water. The DSS-containing drinking water was made up fresh every day to avoid bacterial contamination.

### Disease activity score

During the course of the IAA and DSS treatment, general parameters of animal welfare were carefully monitored every day. Body weight was measured at the beginning and the end of the treatment period and the health status of the animals was evaluated by recording the body weight and by calculating a disease activity score (DAS) at the end of the 7-day treatment period. This score covers fur appearance (score 0: normal, score 1: disturbed), stool consistency (score 0: normal, score 1: soft but formed stool, score 2: loose stool) and presence of blood in the perianal region (score 0: no trace of blood, score 1: traces of blood in perianal region, score 2: bloody perianal region). The scores in each category were summed up, resulting in a total score between 0 and 5.

### Water avoidance stress (WAS)

The WAS procedure represents a psychological stress paradigm. To this end, mice were placed on a small platform (6×3×6 cm, length × width × height) in the centre of a water-filled tank (61×40×22 cm, length × width × height), the level of the water (25°C) in the tank being 0.5 to 1 cm below the platform. The stress procedure was carried out in a brightly lit room (230–250 lux). Following exposure to WAS for 30 min the animals were returned to their home cage.

### Morris water maze (MWM) test

The Morris water maze (MWM) test is a task to evaluate spatial learning and memory [46]. The MWM consisted of a hidden escape platform in an open circular tank made of black plastic material (diameter: 120 cm, depth: 60 cm) which was filled approximately half with water of 25–26°C temperature. The water in the tank was made opaque by addition of white non-toxic tempera paint (Rhoximat SB112, Rhodia, Paris, France). The task of a mouse placed in the water tank was to find the escape platform (diameter: 10 cm) that was submerged 1 cm below the water surface and positioned half-way between the centre and the wall of the tank. Visual cues were placed around the pool in plain sight of the animals [47], [48]. When released into the pool, the mouse swam around in search of an exit while the track of the movements and the time taken to reach the platform (latency) were recorded with the software VideoMot2 (TSE Systems, Bad Homburg, Germany). The light intensity on the water surface in the tank was 50–80 lux.

**Figure 1:**
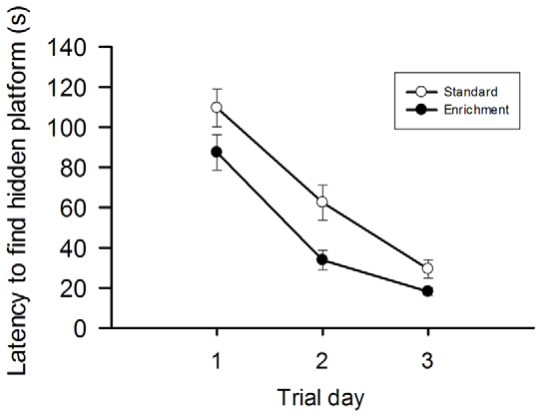
Environmental enrichment enhances spatial learning and memory in the Morris water maze. The graph displays the effects of environmental enrichment, relative to standard housing, for 9 weeks on the behaviour of mice in this task. Mice were tested for 3 consecutive days (6 trials per day) at the end of the respective housing period. The average latency to find the hidden platform on each trial day is shown. The values represent means ± SEM, n = 10.

Following their transfer into the tank, the mice were allowed to swim for a maximum time of 3 min to locate the platform. Once the mice had located the platform, they were quickly picked up and allowed to warm up and dry off under an infrared lamp Those who failed to find the platform within the allotted time were picked up and placed on the platform to rest there for 20 s and assigned a latency of 180 s. Afterwards they were also placed under an infrared lamp. Once the mice were dry, they were returned to their home cage. The entire MWM test procedure took three consecutive days, each animal being subjected to 6 trials per day [47], [48] with a minimum inter-trial interval of 30 min (Figure 1).

### Myeloperoxidase (MPO) levels in the stomach and colon

The tissue levels of MPO were used to quantify inflammation-associated infiltration of neutrophils and monocytes into the tissue [49]. After euthanasia, full-thickness specimens of the gastric corpus and distal colon were excised, shock-frozen in liquid nitrogen and stored at −70°C until assay. After weighing, the frozen tissues were placed, at a ratio of 1 mg: 0.02 ml, in MPO lysis buffer. The composition of this buffer was: 200 mM NaCl, 5 mM ethylenediaminetetraacetic acid, 10 mM trishydroxymethylaminomethane, 10% glycine, 0.1 mM phenylmethylsulphonyl fluoride, 1 µg/ml leupeptide, 28 µg/ml aprotinin, pH 7.4. The samples were homogenized on ice with an Ultraturrax (IKA, Staufen, Germany) and then subjected to two centrifugation steps at 6,000×g (7,000 rpm) and 4°C for 15 min. The MPO (donor: H_2_O_2_ oxidoreductase, EC 1.11.1.7) content of the supernatant was measured with an enzyme-linked immunosorbent assay kit specific for the rat and mouse protein (Hycult Biotechnology, Uden, The Netherlands). The sensitivity of this assay is 1 ng/ml at an intra- and inter-assay variation of around 10%.

### Circulating corticosterone

Trunk blood was collected 2 h after the beginning of the 30-min exposure to WAS to measure post-stress levels of corticosterone. Since the concentration of circulating corticosterone is subject to circadian variations, blood was sampled between 10∶00 h and 12∶00 h. To this end, the mice were deeply anaesthetized with intraperitoneal pentobarbital (150 mg/kg) before they were decapitated. Within 2 min after the injection of the anaesthetic, trunk blood was collected into vials coated with ethylenediamine tetraacetate (Greiner, Kremsmünster, Austria) kept on ice. Following centrifugation for 10 min at 4°C and 1200×g, blood plasma was collected and stored at −70°C until assay. The plasma levels of corticosterone were determined with an enzyme immunoassay kit (Assay Designs, Ann Arbor, Michigan, USA). According to the manufacturer's specifications, the sensitivity of the assay is 27 pg/ml, and the intra- and inter-assay coefficient of variation amounts to 7.7 and 9.7%, respectively.

### c-Fos immunohistochemistry

**Figure 2:**
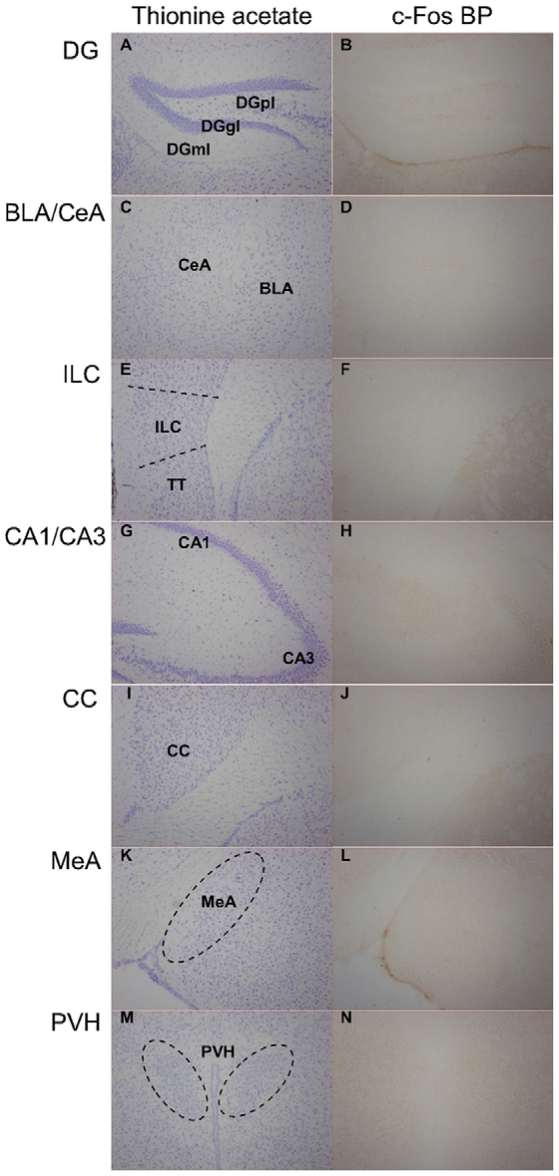
Pre-absorption of the c-Fos antibody with c-Fos blocking peptide prevents c-Fos staining in all brain regions under study. The left column (panels A, C, E, G, I, K, M) shows representative micrographs of the brain regions stained with thionine acetate to reveal their morphology. Parallel sections processed for c-Fos immunocytochemistry in which the c-Fos antibody was pre-absorbed with excess c-Fos blocking peptide (BP) are shown in the right column (panels B, D, F, H, J, L, N). No specific c-Fos staining was detected in any of the brain regions when the pre-absorbed antibody was used. Abbreviations: BLA  =  Basolateral amygdala; CA1  =  CA1 field of the hippocampus; CA3  =  CA3 field of the hippocampus; CC  =  Cingulate cortex; CeA  =  Central amygdala; DGgl  =  Dentate gyrus, granular cell layer; DGml  =  Dentate gyrus, molecular cell layer; DGpl  =  Dentate gyrus, polymorph cell layer; EE  =  Enriched environment; ILC  =  Infralimbic cortex; MeA  =  Medial Amygdala; PVH  =  Paraventricular nucleus of the hypothalamus; SE  =  Standard environment; TT  =  Tenia tecta.

The activation of neurons in select nuclei and cortical areas of the brain was visualized by c-Fos immunocytochemistry 90 min after exposure of the animals to WAS for 30 min. Following euthanasia the brains were removed, frozen in 2-methylbutane (Carl Roth, Karlsruhe, Germany) on dry ice and stored at −70°C until use. Immunocytochemistry was performed according to a slightly modified version of the protocol provided by Sundquist et al. [50]. Serial coronal sections of 20 µm thickness were cut from each forebrain with a cryostat, mounted on Superfrost Plus slides (Menzel, Braunschweig, Germany) and stored at −20°C. Every sixth section was used for c-Fos immunocytochemistry. The sections were surrounded with a hydrophobic barrier pen (ImmEdge Pen, Vector Laboratories, Burlingame, California, USA) and then incubated for 10 min in 4% paraformaldehyde (Sigma-Aldrich, Vienna, Austria) in 0.1 M phosphate-buffered saline (PBS) of pH 7.4. Afterwards the slides were washed three times for 5 min in washing buffer (WB; 0.1 M PBS with 0.05% Tween 20) and incubated in 0.3% H_2_O_2_ for 15 min. After three further washes in WB, the tissues were incubated with 10% goat serum in antibody diluent (AD; 0.1 M PBS containing 0.05% Tween 20 and 1% bovine serum albumin) for 5 min and then with the primary antibody in AD (rabbit polyclonal anti-c-Fos SC-52, 1: 2,000, Santa Cruz Biotech, Santa Cruz, California, USA) overnight at 4°C. On the next day the sections were washed three times in WB and incubated for 30 min in AD containing the biotinylated secondary antibody (goat anti-rabbit IgG 1∶200, Vectastain Elite ABC Kit, Vector Laboratories) at room temperature. After three further washes in WB they were incubated for 30 min in avidin-biotin complex (Vectastain Elite ABC Kit, Vector Laboratories). Subsequently the tissues were rinsed three times in WB and developed with 3,3-diaminobenzidine substrate (DAB substrate kit for peroxidase, Vector Laboratories). Finally the sections were washed three times for 5 min in distilled water, air-dried overnight, cleared in 100% xylol and coverslipped with Entellan (Merck, Darmstadt, Germany). Antibody specificity was tested by pre-absorbing the primary antibody with an excess amount of c-Fos blocking peptide (SC-52P, Santa Cruz Biotech, Santa Cruz, California, USA). Following this procedure no specific staining was detectable in any brain region under study (Figure 2).

### Cell counting and quantification

The immunocytochemically processed brain sections were examined with a light microscope (Axiophot, Zeiss, Oberkochen, Germany) coupled to a computerized image analysis system (MCID Basic, version 7.0, Imaging Research Inc., Brock University, St. Catharines, Ontario, Canada). The slides were coded such that the investigator was blind to the treatment group under investigation. Brain regions of interest (ROIs) were identified with the help of adjacent Nissl-stained sections and the mouse brain stereotaxic atlas of Paxinos and Franklin [51]. In order to count all c-Fos positive cells (cells containing a brown/black reaction product of sufficient intensity within the nucleus) with the computerized image analysis system, an intensity-based background threshold was determined for each brain ROI. The background threshold was defined such that the maximum number of c-Fos labelled cells was counted without inclusion of any background staining. While in the granular cell layer of the dentate gyrus and the paraventricular nucleus of the hypothalamus all c-Fos positive cells were counted, the number of c-Fos labelled cells in the other ROIs was quantitated within a square of 200×200 µm.

Three consecutive sections were counted bilaterally to quantitate the number of c-Fos positive cells in the cingulate cortex (Bregma +1.10 to +0.86), the basolateral, central and medial amygdala (Bregma −1.06 to −1.34) as well as the CA1 field, the CA3 field and the granular cell layer of the dentate gyrus of the hippocampus (Bregma −1.34 to −1.58). Two consecutive sections were counted bilaterally to evaluate the expression of c-Fos in the infralimbic cortex (Bregma +1.54 to +1.42) and the paraventricular nucleus of the hypothalamus (Bregma −0.58 to −0.70). The cell counts obtained for each ROI in the different sections of each animal were averaged to quantitate the mean number of c Fos-positive cells within a particular brain region of that animal. These average values/brain region of each animal were used for statistical analysis.

### Statistics

Statistical evaluation of the results was performed on SPSS 18.0 (SPSS Inc., Chicago, IL, USA). The data were analyzed by two-way analysis of variance (ANOVA). The homogeneity of variances was assessed with the Levene test. Post-ANOVA analysis of group differences was performed with the Tukey HSD (honestly significant difference) test, when the variances were homogeneous, and with the Games-Howell test, when the variances were unequal. Student's t-test was used when only two groups were compared. In view of the exploratory nature of the study, probability values ≤0.1 [52]–[54] were regarded as statistically significant. All data are presented as means ± SEM, n referring to the number of mice in each group.

## Results

### Enriched housing improved spatial learning and memory in the Morris water maze (MWM) test

Spatial learning and memory was assessed with the MWM test in which the escape from the water to a hidden platform reinforces the animal's desire to quickly find the platform. As shown in Figure 1, the mice learned to locate the hidden platform more quickly on repeated trials, and the performance in this spatial learning and memory task was significantly enhanced when the animals were kept under enriched conditions for 9 weeks. Two-way repeated-measures ANOVA showed that the latency to find the hidden platform differed with housing condition (F_(1, 118)_  = 11.804, P = 0.001) and trial day (F_(1.551, 182.959)_  = 63.996, P<0.001), without a significant interaction between these factors.

### Enriched housing increased the susceptibility to DSS-induced colitis, but not IAA-induced gastritis

**Figure 3:**
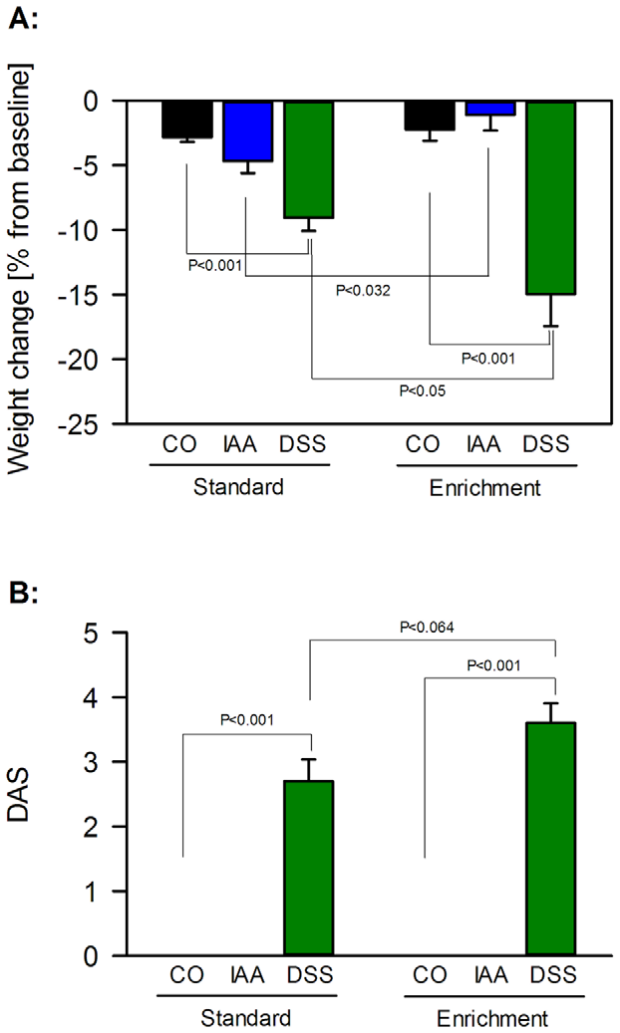
Environmental enrichment increases dextran sulfate sodium (DSS)-induced weight loss and disease severity. Mice were treated for 7 days with iodoacetamide (IAA, 0.1%, added to the drinking water) or DSS (2%, added to the drinking water) or kept under control conditions (CO, plain drinking water) after standard or enriched housing for 9 weeks. Body weight was measured before and after the treatment period, and the weight loss (panel A) during the treatment period was expressed as a percentage of the body weight measured pre-treatment. The disease activity score (DAS, panel B), a measure of the animal's health status, was assessed at the end of the treatment period. The values represent means − SEM (A) and means + SEM (B), n = 10.

At the end of the 7-day treatment with IAA or DSS, the health status of the animals was evaluated by measuring body weight and DAS, while gastrointestinal inflammation was assessed by the MPO content of the stomach and colon, respectively. These parameters differed with housing and were influenced by treatment with DSS and IAA. Thus, the loss of body weight over the treatment period depended on treatment (F_(2,54)_  = 33.215, P<0.001) but not housing, with a significant interaction between these factors (F_(2,54)_  = 6.741, P = 0.002). Specifically, animals treated with DSS lost significantly more weight than the respective control animals under both housing conditions. Furthermore, DSS-treated animals kept under EE lost significantly more weight than DSS-treated mice under standard housing, while IAA-treated animals kept under EE lost less weight than IAA-treated mice under standard housing (Figure 3A). At the end of the treatment period the body weight varied with treatment (F_(2,54)_  = 9.546, P<0.001) and housing (F_(1,54)_  = 13.597, P<0.001) without a significant interaction between these factors (Table 2).

**Table 2:** Body weight (g) at the end of the treatment period.

	Standard Environment	Enriched Environment
**Control**	28.2±0.26	26.9±0.44
**Iodoacetamide**	27.5±0.35	26.9±0.38
**Dextran sulphate sodium**	26.8±0.71	24.0±0.78

Means ± SEM, n = 10 per group.

Treatment with DSS, but not IAA, was associated with symptoms of colonic inflammation such as a bloody perianal region and soft to loose stool consistency. These symptoms were quantified by the DAS which varied with housing conditions (F_(1,54)_  = 3.941, P = 0.052) and treatment (F_(2,54)_  = 193.086, P<0.001), with a significant interaction between these factors (F_(2,54)_  = 3.941, P = 0.025). Figure 3B shows that the DAS in DSS-treated mice was larger under enriched housing than under standard housing.

**Figure 4:**
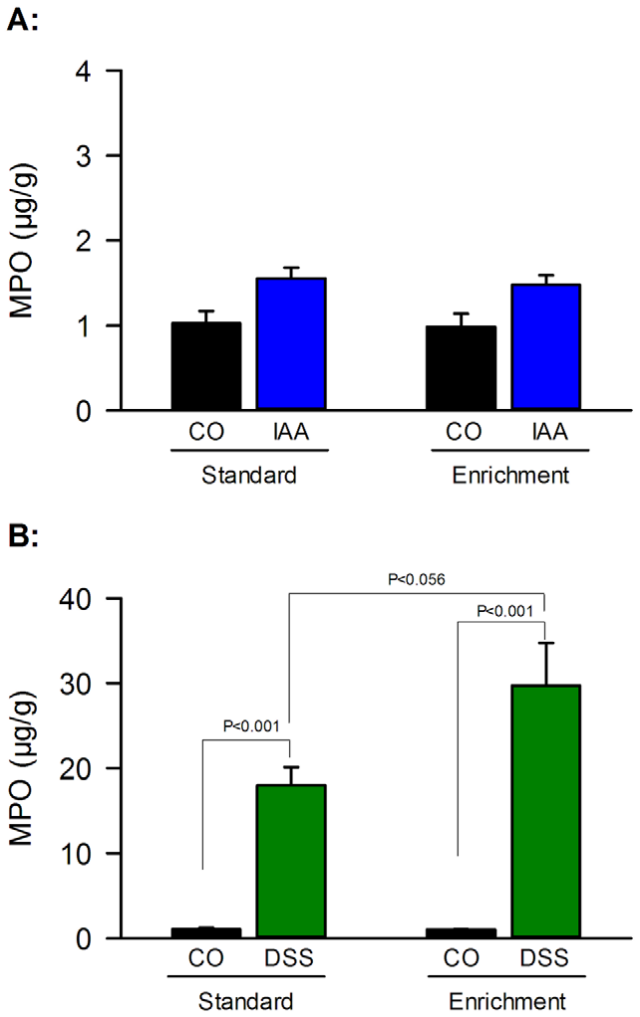
Environmental enrichment increases the dextran sulfate sodium (DSS)-induced rise of colonic myeloperoxidase (MPO) content, but does not influence the iodoacetamide (IAA)-induced rise of gastric MPO content. Mice were treated for 7 days with IAA (0.1%, added to the drinking water) or DSS (2%, added to the drinking water) or kept under control conditions (CO, plain drinking water) after standard or enriched housing for 9 weeks. The gastric (panel A) and colonic (panel B) MPO content was determined at the end of the treatment period. Note that the scale of the ordinate is different between panel A and B. The values represent means + SEM, n = 10.

The gastric and colonic MPO content was used as a measure of inflammation induced by IAA and DSS, respectively. IAA treatment increased the gastric MPO content under standard and enriched housing conditions to a similar extent (Figure 4A). As shown by two-way ANOVA, the gastric MPO content varied with treatment (F_(1,36)_  = 14.411, P = 0.001), but not with housing conditions, without a significant interaction between these factors (Figure 4A). In contrast, the DSS-evoked increase of the colonic MPO content was larger under enriched than under standard housing (Figure 4B). Specifically, the colonic MPO content varied with housing (F_(1,35)_  = 5.256, P = 0.028) and treatment (F_(1,35)_  = 80.107, P<0.001), with a significant interaction between these factors (F_(1,35)_  = 5.319, P = 0.027).

### Colitis enhanced the stress-induced rise of plasma corticosterone under enriched, but not standard housing

**Figure 5:**
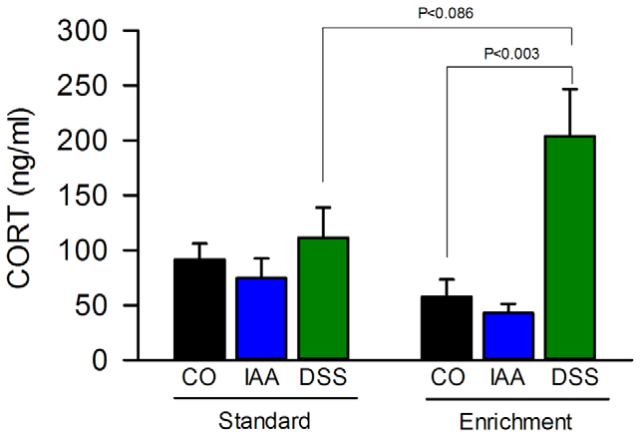
Environmental enrichment increases the dextran sulfate sodium (DSS)-induced rise of post-stress corticosterone (CORT) levels. Mice were treated for 7 days with IAA (0.1%, added to the drinking water) or DSS (2%, added to the drinking water) or kept under control conditions (CO, plain drinking water) after standard or enriched housing for 9 weeks. Plasma CORT was measured 2 h after the beginning of a 30-min exposure to water avoidance stress at the end of the treatment period. The values represent means + SEM, n = 10.

Plasma corticosterone was determined 2 h after the beginning of a 30 min exposure to WAS, i.e., at the time when the brain was removed for visualization of c-Fos expression. Two-way ANOVA showed that post-stress corticosterone depended on treatment (F_(2,54)_  = 9.955, P<0.001) but not housing, with a significant interaction (F_(2,54)_  = 4.609, P = 0.014) between both factors. Further analysis disclosed that, relative to the values measured in control animals, treatment with DSS caused a significant rise of the plasma corticosterone levels under enriched, but not standard housing, whereas IAA had no effect (Figure 5).

### Enriched housing enhanced the stress-induced c-Fos expression in the dentate gyrus and unmasked an inhibitory effect of gastritis and colitis

**Figure 6:**
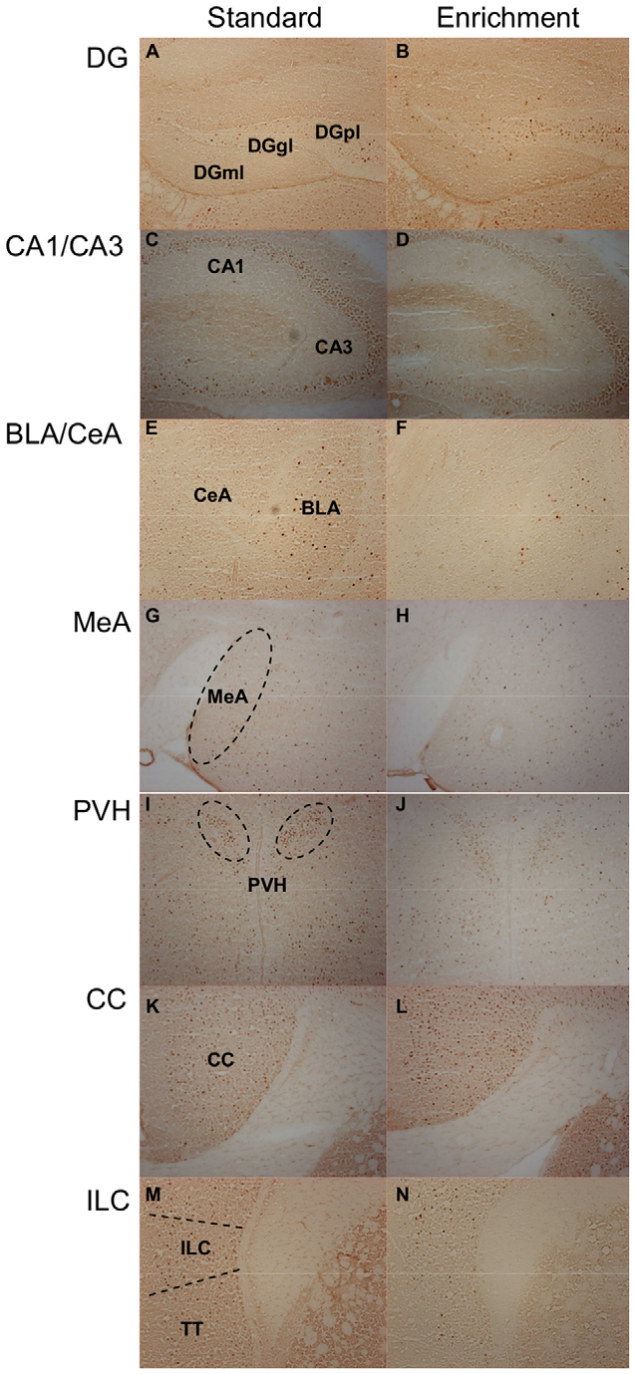
Environmental enrichment alters stress-induced c-Fos expression in the forebrain. The effect of water avoidance stress (30 min) on the cerebral c-Fos expression was examined in mice kept for 9 weeks under standard (SE) or enriched environment (EE). The left column panels (A, C, E, G, I, K, M) show representative micrographs of 9 forebrain regions taken from SE mice while the right column panels (B, D, F, H, J, L, N) depict micrographs of the same brain regions taken from EE mice. Relative to SE animals, EE increased stress-induced c-Fos expression in the granular cell layer of the dentate gyrus (A, B), but decreased it in the CA1 region of the hippocampus (C, D), the central amygdala (E, F) and the infralimbic cortex (M, N). In contrast, EE failed to modify stress-induced c-Fos expression in the CA3 region of the hippocampus (C, D), the basolateral and medial amygdala (E, F, G, H), the paraventricular nucleus of the hypothalamus (I, J) and the cingulate cortex (K, L). Abbreviations: BLA  =  Basolateral amygdala; CA1  =  CA1 field of the hippocampus; CA3  =  CA3 field of the hippocampus; CC  =  Cingulate cortex; CeA  =  Central amygdala; DGgl  =  Dentate gyrus, granular cell layer; DGml  =  Dentate gyrus, molecular cell layer; DGpl  =  Dentate gyrus, polymorph cell layer; ILC  =  Infralimbic cortex; MeA  =  Medial Amygdala; PVH  =  Paraventricular nucleus of the hypothalamus; TT  =  Tenia tecta.

**Figure 7:**
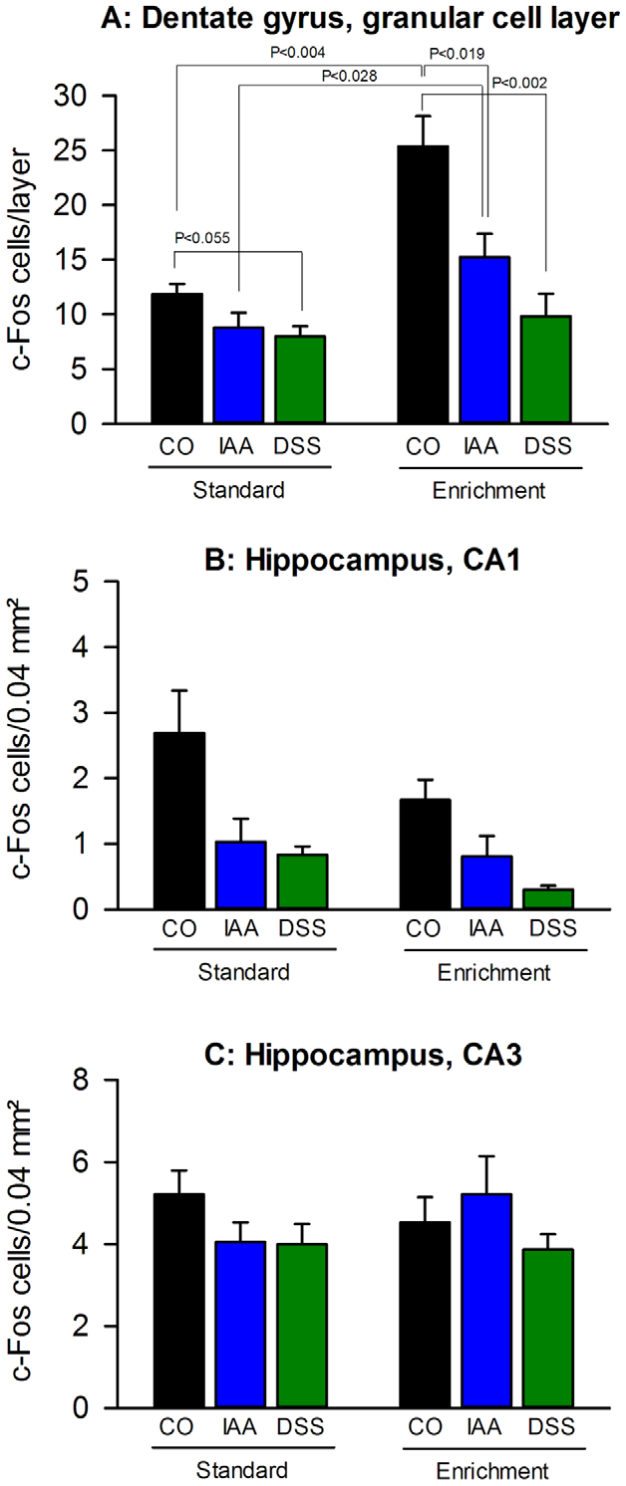
Environmental enrichment and visceral inflammation modify stress-induced c-Fos expression in the hippocampus. Environmental enrichment increases stress-induced expression of c-Fos in (A) the granular cell layer of the dentate gyrus, decreases c-Fos expression in (B) the hippocampal CA1 region, but does not alter c-Fos levels in (C) the hippocampal CA3 region. Gastritis evoked by iodoacetamide (IAA) and colitis evoked by dextran sulphate sodium (DSS) dampen stress-induced c-Fos expression in (A) the dentate gyrus and (C) the hippocampal CA1 region of mice kept both under standard (SE) or enriched environment (EE). Mice were maintained for 9 weeks under SE or EE and then treated for 7 days with IAA (0.1% added to the drinking water) or DSS (2% added to the drinking water), while control (CO) mice drank plain water. Expression of c-Fos was visualized 2 h after the beginning of a 30-min exposure to water avoidance stress at the end of the treatment period. The values represent means + SEM, n = 6–7.

The number of cells expressing c-Fos in the granular cell layer of the dentate gyrus (DGgl) (Figures 6A, B and 7A) and in the CA1 region (Figures 6C, D and 7B) of the hippocampus following exposure to WAS was markedly changed by housing and treatment, whereas the stress-induced expression of c-Fos in the CA3 region (Figures 6C, D and 7C) remained unaltered by housing and treatment (Figure 7C).

The most remarkable effects of the experimental interventions under study were seen in the DGgl, given that housing (F_(1,35)_  = 25.198, P<0.001) and treatment (F_(2,35)_  = 15.324, P<0.001) interacted (F_(2,35)_  = 5.408, P = 0.009) with each other. On the one hand, enriched housing increased the stress-induced expression of c-Fos in control animals, an effect that was also seen in IAA-treated mice (Figure 7A). On the other hand, enriched housing unmasked a strong inhibitory effect of IAA and DSS treatment on the stress-evoked activation of the DGgl (Figure 7A). In contrast, IAA and DSS treatment under standard housing attenuated the c-Fos expression in the DGgl due to WAS only by a small margin (Figure 7A).

The stress-induced c-Fos expression in the CA1 region of the hippocampus (Figure 7B) also varied with housing (F_(1,35)_  = 4.055, P = 0.052) and treatment (F_(2,35)_  = 10.812, P<0.001), without a significant interaction between these factors. In control mice, the WAS-evoked expression of c-Fos in the CA1 region was depressed by enriched housing. Treatment with IAA and DSS blunted the stress-evoked activation of the CA1 region both under standard and enriched housing (Figure 7B).

### Enriched housing inhibited the stress-induced c-Fos expression in the central amygdala and prevented the inhibitory effect of gastritis and colitis

**Figure 8:**
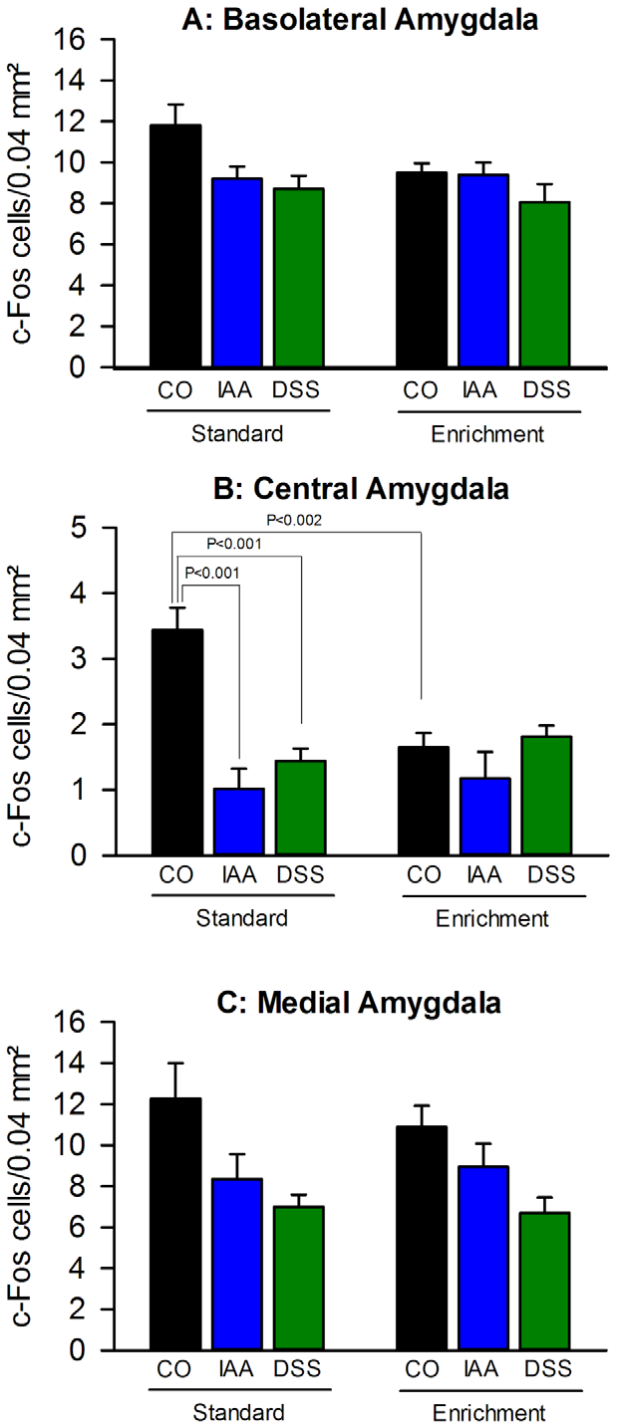
Environmental enrichment and visceral inflammation modify stress-induced c-Fos expression in the amygdala. Environmental enrichment decreases stress-induced c-Fos expression in (B) the central amygdala but does not alter stress-induced c-Fos levels in (A) the basolateral and (C) medial amygdala. Gastritis evoked by iodoacetamide (IAA) and colitis evoked by dextran sulphate sodium (DSS) dampen stress-induced c-Fos expression in all 3 amygdalar nuclei of mice kept under standard environment (SE), an effect that is largely absent in mice kept under enriched environment (EE). Mice were maintained for 9 weeks under SE or EE and then treated for 7 days with IAA (0.1% added to the drinking water) or DSS (2% added to the drinking water), while control (CO) mice drank plain water. Expression of c-Fos was visualized 2 h after the beginning of a 30-min exposure to water avoidance stress at the end of the treatment period. The values represent means + SEM, n = 7–8.

The number of cells expressing c-Fos in the basolateral amygdala (BLA), central amygdala (CeA) and medial amygdala (MeA) in response to WAS differed with treatment and/or housing conditions (Figures 6E, F, G, H and 8A, B, C). In the BLA, the stress-induced expression of c-Fos was unrelated to housing but differed with treatment (F_(2,37)_  = 4.753, P = 0.015), without an interaction between these factors. The treatment effect was particularly evident under standard housing where stress-evoked activation of the BLA in both IAA- and DSS-treated mice was less than that in control animals (Figure 8A).

The stress-induced expression of c-Fos in the CeA (Figures 6E, F and 8B) varied with housing (F_(1,37)_  = 3.178, P = 0.083) and treatment (F_(2,37)_  = 13.080, P<0.001), with a significant interaction between these factors (F_(2,37)_  = 8.676, P = 0.001). Under standard housing, the stress-evoked activation of the CeA was markedly depressed by IAA and DSS treatment. This treatment effect was absent under EE which in control animals itself depressed stress-induced c-Fos expression by about 50% (Figure 6E, F).

The number of cells expressing c-Fos in the MeA in response to WAS did not differ when the animals were housed under standard or enriched conditions, while treatment (F_(2,37)_  = 8.357, P = 0.001) with DSS and IAA reduced the stress-induced expression of c-Fos both under standard and enriched housing (Figure 6G, H and 8C).

### Enriched housing inhibited the stress-induced c-Fos expression in the infralimbic cortex and attenuated the inhibitory effect of gastritis and colitis but had little effect in the cingulate cortex and hypothalamus

**Figure 9:**
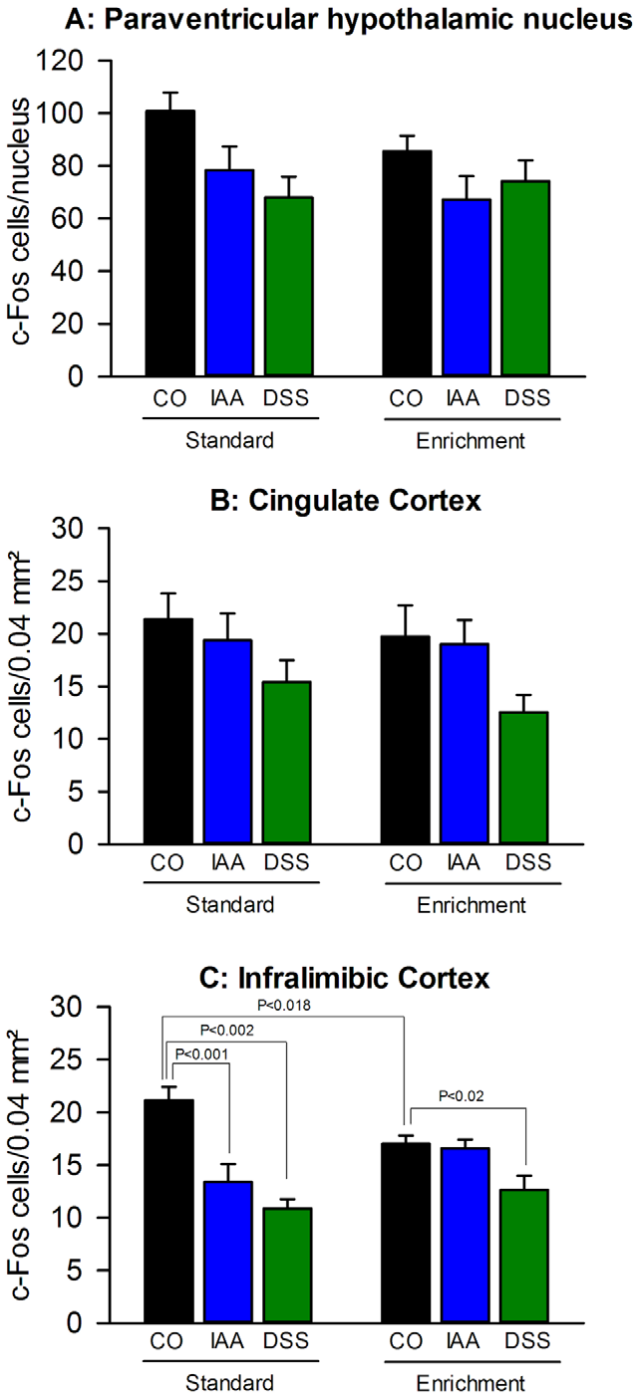
Environmental enrichment and visceral inflammation modify stress-induced c-Fos expression in the medial prefrontal cortex and the hypothalamus. Environmental enrichment decreases stress-induced c-Fos expression in (C) the infralimbic cortex but does not alter stress-induced c-Fos levels in (B) the cingulate cortex and (A) paraventricular nucleus of the hypothalamus. Gastritis evoked by iodoacetamide (IAA) and colitis evoked by dextran sulphate sodium (DSS) dampen stress-induced c-Fos expression in mice kept under standard environment (SE) in a region-dependent manner, an effect that is largely absent in mice kept under enriched environment (EE). Mice were maintained for 9 weeks under SE or EE and then treated for 7 days with IAA (0.1% added to the drinking water) or DSS (2% added to the drinking water), while control (CO) mice drank plain water. Expression of c-Fos was visualized 2 h after the beginning of a 30-min exposure to water avoidance stress at the end of the treatment period. The values represent means + SEM, n = 7–8.

The stress-induced c-Fos expression in the paraventricular nucleus of the hypothalamus (PVH) did not significantly depend on housing conditions but was attenuated by IAA and DSS treatment (F_(2,36)_ = 4.742; P = 0.015), particularly under standard housing (Figures 6I, J and 9A). There was no significant interaction between the two factors. Likewise, the WAS-evoked activation of the cingulate cortex (CC) was independent of housing conditions but reduced by DSS treatment (Figures 6K, L and 9B) under both standard and enriched housing (F_(2,37)_  = 4.278, P = 0.021), without a significant interaction between these factors.

Appreciable treatment effects (F_(2,37)_  = 19.324, P<0.001) but no housing effects, with a significant interaction between both factors (F_(2,37)_  = 5.454, P = 0.008), were observed in the infralimbic cortex (ILC). Thus, the number of cells expressing c-Fos in response to WAS in control mice was reduced by enriched housing (Figures 6M, N and 9C). Moreover, treatment with IAA and DSS reduced the stress-evoked activation of the ILC under standard housing, whereas under enriched housing only treatment with DSS blunted the stress-induced c-Fos response (Figure 9C).

## Discussion

### Rationale of the study

EE is enforced as a measure to increase the welfare of captive animals. By enabling them to express a variety of species-specific behaviours, EE is thought to enhance the translational value of animal models in the preclinical study of biological processes relevant to human health and disease [1]. This argument raises the question in which way the biology of laboratory animals is modified by standard versus enriched housing. The present study focused on the cerebral stress response of mice kept under enriched housing, given that EE is considered to reduce the stress which impoverished maintenance under standard conditions imposes on the animals [55]. In line with this contention, Belz et al. [17] report that the basal levels of circulating corticosterone in rats are lowered when the animals are kept under enriched housing, while other studies have failed to confirm this finding [4], [18], [20], [41].

Against this background we set out to record the activation of stress-relevant brain nuclei in response to WAS, a psychological stress paradigm, and examine whether this brain response is altered by EE. In addition, we explored whether the brain response to WAS is modified by internal stressors such as inflammation which causes long-term alterations in pain sensitivity [21]–[23] and anxiety [45], [56]. We hypothesized that inflammation could reset the cerebral sensitivity to psychological stress and that this interaction is modulated by EE. The enriched housing procedure used here incorporated elements of published protocols [42]–[44], and the duration of enriched housing (9 weeks) was modelled along the studies of Marashi et al. [41] and Amaral et al. [57] who found that 8 weeks of enriched housing have a particularly profound impact on behaviour. The effect of the current protocol to foster spatial learning and memory through increasing neuronal plasticity in the brain [1]–[3] was confirmed by the improved MWM performance of the mice kept under EE.

### Influence of environmental enrichment on stress-induced c-Fos expression in the brain

Neuronal activation as visualized by c-Fos-like immunoreactivity was assessed in cerebral regions known to be involved in the general stress response, including the hypothalamus, hippocampus, amygdala, infralimbic and cingulate cortex [24], [58]–[60]. As previously reported for various stress paradigms [24], [58]–[62], WAS caused expression of c-Fos in all regions under study, while c-Fos expression under basal (non-stress) conditions was very low, at least in the extra-cortical regions of the brain.

EE led to a significant change in the WAS-evoked c-Fos expression in 4 brain regions: the DGgl, the CA1 region of the hippocampus, the CeA and the ILC. While in the ILC, CeA and CA1 region EE attenuated the stress-induced expression of c-Fos, the opposite was true in the DGgl in which EE enhanced the number of c-Fos-expressing neurons. An increase in c-Fos expression within a brain region is thought to represent increased afferent input to and/or increased external stimulation of this region rather than increased depolarization of neurons [63], [64]. We hypothesize that changes in stress-evoked c-Fos expression reflect changes in stress-mediated stimulation of neurons caused by the housing and treatment conditions under study. The effects of EE on c-Fos expression within the DGgl are consistent with other effects of EE in the dentate gyrus. For instance, various EE paradigms stimulate adult neurogenesis [19], [65]–[67] which in the dentate gyrus takes place in the subgranular zone, the new neurons being subsequently integrated into the granular layer [12], [68], [69]. EE-induced increases in neurotrophin levels, increases in dendritic growth and other morphological changes as well as alterations in neurotransmitter dynamics have also been observed in the dentate gyrus [2], [13], [70]. Importantly, the current findings indicate that the structural and neurochemical effects of EE result in alterations of stress-induced activity in the dentate gyrus.

In contrast to what was seen in the DGgl, the stress-induced c-Fos expression in the CA1 region of the hippocampus, the CeA and the ILC region of the medial prefrontal cortex was decreased by EE. The similarity of the effect of EE in these regions may be explained by the close connections between CA1, CeA and ILC [71]–[75]. The CeA is a major output nucleus of the amygdala to autonomic brainstem centres and endocrine regions of the hypothalamus [76]–[78] and receives input from the CA1 region, while there are no monosynaptic connections with the dentate gyrus [71]. Like the hippocampus, the amygdala is involved in the processing of stress and emotions such as fear [79]. The attenuation of stress-induced stimulation of the CeA under enriched housing may hence imply that EE reduced the fear perceived during the WAS procedure in a novel environment. This argument is supported by findings that animals kept under EE adapt more quickly to novel situations and can better cope with stress than animals kept under standard conditions [20], [80]–[82]. These behavioural adaptations may also be related to the effect of EE to blunt the stress-induced stimulation of neurons in the ILC, an observation that is in line with the ability of EE to dampen stress-induced acetylcholine and dopamine release in the prefrontal cortex [83], [84].

The divergent effects of EE on WAS-evoked c-Fos expression in the DGgl, on the one hand, and the ILC, CeA and CA1 region, on the other hand, need to be seen in context with the reciprocal connections between amygdala, hippocampus and prefrontal cortex [58], [71], [73], [74], [85]. The hippocampus and amygdala are essential components of the cerebral circuitry mediating the stress response. Importantly, the two structures issue opposing outputs to the stress effector system [58], [73], [86], [87]. The amygdala (including the CeA) activates the behavioural and endocrine centres of the stress response [76], while the hippocampus provides an important negative feedback on the stress effector system [88]. Since EE reduced the stress-induced stimulation of the CeA but enhanced it in the DGgl, it would appear that EE reduced the overall activity of the stress effector system by altering the impact of the amygdala-hippocampus network on this system. In other terms, EE improved stress resilience. This conclusion is in keeping with a number of observations that EE is able to counteract and prevent the behavioural and neuroendocrine effects of stress [4], [6], [17]–[20], [65], [89], [90].

### Influence of environmental enrichment on gastrointestinal inflammation

The impact of gastrointestinal inflammation on the cerebral stress response to WAS under standard and enriched housing was studied by the use of IAA-induced gastritis [26], [27] and DSS-induced colitis [28], [29]. Both models of inflammation are convenient to handle because IAA and DSS are added to the drinking water and the degree of inflammation can be titrated by the concentration of IAA and DSS in the drinking water. The severity of inflammation was estimated by the increase in the MPO levels in the gastric and colonic wall, respectively [29], [56], which reflect inflammation-associated infiltration of neutrophils and monocytes into the tissue [49]. Previous experiments had established that the gastritis evoked by 0.1% IAA and the colitis evoked by 2% DSS are mild and do not cause any substantial deterioration of mucosal architecture [29], [45]. The induction of colitis was, in addition, confirmed by a rise of DAS which relates to the overall health status of the animals. As judged from the increase in tissue MPO levels, the degree of gastric inflammation appeared to be lower than the degree of colonic inflammation. However, we avoided to increase the concentration of IAA in the drinking water beyond 0.1% because, unlike DSS [29], IAA reduces the intake of water [45], which is a confounding factor in this inflammation model.

EE failed to modify IAA-induced gastritis as judged from its lack of effect on DAS and gastric MPO content, whereas the susceptibility to DSS-evoked colitis was enhanced by enriched housing as deduced from an increase in weight loss, DAS and colonic MPO content. The MPO data indicate that EE promotes DSS-induced colitis by enhancing leukocyte infiltration in the colonic tissue [49]. Although it was beyond the scope of this study to analyse this unexpected finding, some mechanistic explanation is offered by reports that EE might increase the activity of the immune system. Thus, there is evidence that EE enhances natural killer cell activity in the spleen [89], causes inconsistent changes in circulating cytokine levels and reduces circulating IgG1 levels [41]. Moreover, EE can promote T-lymphocyte infiltration in a model of viral encephalitis [91]. In view of these findings we hypothesize that DSS-induced colitis is particularly susceptible to the EE-evoked sensitization or stimulation of the immune system, which results in aggravation of inflammation, disease severity and weight loss. It remains to be investigated whether a propensity for inflammation, enhanced locomotion/exploration or reduced food intake explains why mice under EE weighed less than standard-housed mice independently of treatment.

Other factors that could play a role in the EE-induced enhancement of DSS-evoked colitis include the gut microbiota and the enteric nervous system. Inflammatory conditions affect enteric neurons which, in turn, are able to modify intestinal immune responses and epithelial barrier function [92]. DSS is known to enhance colonic mucosal permeability, increase the penetration of bacteria into the intestinal mucus layer, and change gut microbial diversity [93]–[97]. Since these and other environmental factors have an impact on the severity of DSS-induced colitis, great care was taken to avoid contamination of the EE cages by external microbiota. It awaits to be examined whether EE *per se* modifies mucosal permeability or alters the gut microbiota in a way that increases mucosal vulnerability by DSS.

### Influence of gastrointestinal inflammation on stress-induced c-Fos expression in the brain under standard and enriched housing

Under standard housing, experimental gastritis and colitis inhibited stress-induced *c-Fos expression* in several nuclei of the brain, most prominently in the CeA, the CA1 region of the hippocampus and the ILC. The WAS-induced expression of c-Fos in the BLA, MeA, DGgl, CC and PVH was also reduced by gastrointestinal inflammation, albeit to a smaller extent. The effect of DSS-evoked colitis was in general more pronounced than that of IAA-evoked gastritis, which may be related to different degrees of inflammation in the colon and stomach or to region-specific differences in signal transmission from the gut to the brain. The present observations are important inasmuch as they show that an internal (systemic) stressor can modify the cerebral response to an external (psychological) stressor. This finding is analogous to explaining experimental and clinical observations that gastrointestinal inflammation and stress can interact with each other in aggravating gastrointestinal disease, hyperalgesia and anxiety, which are prevalent in patients with inflammatory bowel disease and irritable bowel syndrome [39], [98].

Conceptually, the interaction between inflammation and stress can take place at several levels of the gut-brain axis. Within the gut, WAS has been found to enhance the permeability of the intestinal mucosa [36], [37], which is likely to promote activation of the mucosal immune system and the development of mucositis. Accordingly, stress has been found to aggravate experimentally induced colitis [34] and to reactivate quiescent colitis [32]. Stress and inflammation are also able to cause mechanical and chemical hypersensitivity in the gastrointestinal tract [25]–[29], [35], but an interaction between stress and inflammation in the development of visceral hypersensitivity has been negated [33]. Within the brain it has been shown that colitis upregulates the expression of corticotropin-releasing factor mRNA in the magnocellular part of the PVH but blunts the stress-induced increase in corticotropin-releasing factor transcription in the parvocellular part of the PVH [30].

Enriched housing did not alter the inhibitory effect of gastrointestinal inflammation, notably colitis, on WAS-induced c-Fos expression in the CC, MeA and CA1 region of the hippocampus. In the ILC and CeA, however, the inflammation-evoked inhibition of the c-Fos response to WAS was blunted or abolished by EE, whereas in the DGgl it was greatly amplified. These observations further attest to the ability of environmental factors to modify the complex interactions between internal and external stressors in the prefrontal cortex – amygdala – hippocampus circuitry in a topographically specific manner. The divergent effects which EE exerts in the CeA and DGgl emphasize the opposing roles of the amygdala and hippocampus in the orchestration of the stress response [58], [73], [76], [86]–[88]. It remains to be explored in which way the alterations in the cerebral c-Fos response to WAS under gastrointestinal inflammation and EE translate to alterations in stress coping and emotional behaviour.

### Influence of gastrointestinal inflammation on post-stress levels of circulating corticosterone under standard and enriched housing

The circulating levels of corticosterone were measured when the stress-induced expression of c-Fos in the brain was visualized. Without knowing the basal (pre-stress) corticosterone concentrations it is not possible to deduce the magnitude of the corticosterone response to WAS (which usually is maximal within 20 min after exposure to stress) from the post-stress levels of this glucocorticoid [99]. The post-stress levels of corticosterone measured here provide, however, some information on the time course of the corticosterone response. Under these precautions it can be surmised that colitis, but not gastritis, caused a significant rise and/or prolongation of the corticosterone response to WAS under enriched, but not standard housing. Although circulating corticosterone is thought to be an index of HPA axis activity [41], [89], [100], the elevated corticosterone levels post-stress in mice under conditions of EE and colitis were not associated with an increased expression of c-Fos in the PVH. In assessing this finding it is appropriate to consider that colitis has differential effects on the magnocellular and parvocellular part of the PVH and can even blunt the corticosterone response to WAS [30]. Given that an appropriate HPA axis response to internal and external threats, as investigated in the present study, is crucial for homeostasis [101], the enhanced post-stress increase of corticosterone under conditions of EE and colitis can be seen as beneficial effect: corticosterone would not only counteract inflammation but also exert a regulatory feedback effect on the cerebral stress circuitry [73], [76].

### Conclusion

Visualization of neuronal activation in the cerebral stress circuitry by c-Fos expression revealed a complex interaction between external stress (WAS), internal stress (gastrointestinal inflammation) and environmental (housing) conditions. Four major findings evolved. (1) EE had region-specific effects on WAS-induced c-Fos expression in the prefrontal cortex – amygdala – hippocampus network. Specifically, EE enhanced stress-induced c-Fos expression in the DGgl but reduced it in the CeA. This observation indicates that EE alters the overall impact of the amygdala – hippocampus network on the stress effector system and thus improves stress resilience. (2) EE aggravated DSS-evoked colitis, which is explained by a sensitizing or stimulant effect of EE on the immune system, causing aggravation of the inflammatory process. (3) Colitis inhibited stress-induced c-Fos expression in several nuclei of the brain, most prominently in the CeA, the CA1 region of the hippocampus and the ILC. These observations show that an internal (systemic) stressor modifies the cerebral response to an external (psychological) stressor. (4) EE prevented the colitis-evoked inhibition of the c-Fos response to WAS in the ILC and CeA but amplified it in the DGgl. These observations underscore the ability of environmental factors to modify the complex interaction between internal and external stressors in the cerebral stress circuitry. The current findings also have a bearing on the interaction of inflammation and stress in aggravating gastrointestinal disease and disturbing mental health.
